# Reporting patterns of suicide- and self-injury-related events involving liraglutide, semaglutide, and tirzepatide: data from the European pharmacovigilance database

**DOI:** 10.3389/fphar.2026.1882225

**Published:** 2026-08-27

**Authors:** Cristina Scavone, Michele Cerasuolo, Mario Gaio, Nicoletta Lettera, Francesca Futura Bernardi, Annalisa Capuano, Barbara Rinaldi

**Affiliations:** 1 Department of Experimental Medicine, University of Campania “L. Vanvitelli”, Naples, Italy; 2 Campania Regional Centre for Pharmacovigilance and Pharmacoepidemiology, Naples, Italy; 3 Department of Life Science, Health, and Health Professions, Link Campus University, Roma, Italy; 4 Division of Human Anatomy – Neuronal Networks Morphology and Systems Biology Lab, Department of Mental, Physical Health and Preventive Medicine, University of Campania “Luigi Vanvitelli”, Naples, Italy; 5 Coordination of the Regional Health System, General Directorate for Health Protection, Naples, Italy

**Keywords:** diabetes, eudravigilance, GLP-1 and GLP-1/GIP receptor agonists, obesity, pharmacovigilance, suicide

## Abstract

**Introduction:**

Glucagon-like peptide-1 (GLP-1) and GIP receptor agonists (RAs) are increasingly used for treating type 2 diabetes mellitus and for chronic weight management. As their use expands, more attention is being paid to potential safety concerns, especially psychiatric adverse events. So far, European and United States regulatory agencies have not confirmed a causal relationship between these drugs and suicidality. Continued post-marketing monitoring remains warranted, given their growing use for weight management.

**Methods:**

This study aimed to provide an updated pharmacovigilance analysis of suicide or self-injury-related events reported with liraglutide, semaglutide, and tirzepatide. Individual Case Safety Reports listing liraglutide, semaglutide, or tirzepatide as suspected drugs were retrieved from EudraVigilance. The period covered was 1 January 2021 to 31 December 2025. Reports were included if the indication was consistent with type 2 diabetes mellitus or weight management. Suicide and self-injury events were identified using Preferred Terms from the Standardised MedDRA Query “Suicide/self-injury.” Report characteristics were described. Reporting odds ratio (RORs), along with 95% confidence intervals, were calculated to compare the frequency of suicide and self-injury events across the three drugs.

**Results:**

We included 42,941 eligible reports. Most reports involved semaglutide, then tirzepatide and liraglutide. Gastrointestinal disorders were the most frequent adverse events, followed by injury, poisoning and procedural complications, and general disorders and administration site conditions. Suicide/self-injury events made up a small portion, with 37 cases for liraglutide, 141 for semaglutide, and 47 for tirzepatide. Compared with tirzepatide, the reporting frequency of these events was higher for liraglutide (ROR 2.54, 95% CI 1.60–4.01) and semaglutide (ROR 2.69, 95% CI 1.91–3.83).

**Conclusions:**

These findings do not establish a causal link between GLP-1 and GLP-1/GIP RAs and suicide or self-injury events but simply provide a comparative analysis of disproportionality. Overall, the results do not contradict regulatory conclusions. They should be interpreted with caution, as spontaneous reporting systems have limitations. Continued pharmacovigilance is warranted, especially as use for weight management increases.

## 1 Introduction

Glucagon-like peptide-1 receptor agonists (GLP-1 RAs), which include liraglutide, semaglutide, dulaglutide and exenatide IR/ER, act like the endogenously secreted hormone GLP-1 that is secreted from enteroendocrine L cells lining the intestines following the food intake. These agents increase glucose-stimulated insulin secretion and inhibit glucagon secretion from pancreatic α-cells, correcting postprandial glucose levels. In addition, they decrease gastric emptying rates and stimulate satiety via central mechanisms that directly influence appetite regulation, eventually influencing body weight and caloric intake. Of note, pleiotropy has now been observed with these pharmacological agents, with demonstrated benefits in high-risk subjects that influence lipids, inflammation, and cardiovascular events (Zheng et al., 2024; Alfaris et al., 2024). Recently, GLP-1–based therapies have expanded to include tirzepatide, a compound that acts on both the GIP and GLP-1 receptors; like GLP-1, GIP is an incretin hormone released after meals that enhances glucose-dependent insulin secretion, thereby introducing a “dual-incretin” approach to glucose regulation. These therapies are used to treat type 2 diabetes mellitus (T2DM) and chronic weight management in obese individuals aged over 18 years (BMI ≥ 30 kg/m^2^) and in overweight subjects (BMI ≥ 27 kg/m^2^) with weight-associated co-morbidities, in the context of a comprehensive care program (France and Syed, 2024; Moiz et al., 2025; Singh et al., 2024).

Safety has become increasingly important as exposure has risen sharply, particularly among patients treated for obesity (American Diabetes Associa tion Professional Practice Committee for Diabetes*, 2026). Across the Summary of Product Characteristics (SmPCs) of liraglutide, semaglutide, and tirzepatide, gastrointestinal adverse events (AEs), such as nausea, vomiting, diarrhoea, and constipation, are the most frequently reported AEs, occurring mainly during dose escalation. Less common AEs such as cholelithiasis, cholecystitis, delayed gastric emptying and intestinal obstruction have also been documented, thereby warranting caution in those patients at risk. Other events like injection-site reactions, worsening of diabetic retinopathy, pancreatitis, acute renal failure, medullary thyroid carcinoma, and non-arteritic ischemic optic neuropathy have also been documented during the post-marketing surveillance period (Yeo et al., 2026; Kunutsor and Seidu, 2026).

Given the growing use of these drugs, pharmacovigilance is particularly important to monitor their safety in real-world settings. This is especially true for GLP-1 and GLP-1/GIP RAs, which are rapidly prescribed to large and heterogeneous populations. Post-marketing surveillance may therefore help detect uncommon or unexpected AEs, explore possible differences among individual agents, and provide useful information to support safer prescribing. A recent pharmacovigilance study integrated SmPC-listed adverse drug reactions with disproportionality analyses across liraglutide, semaglutide, and tirzepatide, showing both common and molecule-specific reporting patterns and identifying reports of AEs not listed in the respective SmPCs (Popa Ilie et al., 2026).

In this larger context of safety issues, the psychiatric safety of GLP-1–based therapies has recently appeared as an emerging research field. Data from clinical trials and epidemiological studies seem to not support a causal association between GLP-1 RAs and suicidality and both the European Medicines Agency (EMA) and the U.S. Food and Drug Administration (FDA) declared that there is no supportive causal association between GLP-1 RAs and suicide attempt and/or thought of suicidality (Update on FDA; Lovell, 2024). However, in a previous study carried out by our research group using data from the EudraVigilance (EV) database we found that suicidal events were mostly reported with semaglutide and liraglutide, which were also associated with significantly higher reporting probabilities compared to other GLP-1 RAs (Ruggiero et al., 2024).

Moreover, available pharmacovigilance data suggest differences in the reported pattern and seriousness of psychiatric adverse events across individual agents. In an EudraVigilance analysis, depression, anxiety, and suicidal ideation were the most frequently reported events, while fatal or life-threatening outcomes were reported with liraglutide and semaglutide but not with tirzepatide; these findings should be interpreted cautiously because of differences in market exposure and reporting patterns (Tobaiqy and Elkout, 2024).

Considering the recent marketing approval of tirzepatide and the clinical significance of suicidal events, the present study aimed to provide an updated analysis on the occurrence of these events during treatment with GLP-1 and GLP-1/GIP RAs, by describing data from Individual Case Safety Reports (ICSRs) retrieved from the EV. We also aimed to estimate the reporting probability of suicide related events following the treatment with liraglutide, semaglutide, and tirzepatide.

## 2 Methods

### 2.1 Data source

Data on ICSRs with liraglutide, semaglutide or tirzepatide as the suspected drug were retrieved from the EV database that is managed by the EMA and it is used for the management and collection of ICSRs related to medicines or vaccines, which are authorized or are being studied in clinical trials in the European Economic Area (EEA). The EV contains all ICSRs reported by a healthcare professional (HCP) or a non-HCP to a European Union national competent authority or a marketing authorisation holder. These data are publicly available for transparency through the EMA website (www.adrreports.eu).

### 2.2 ICSR selection with Line Listing

The Medical Dictionary for Regulatory Activities (MedDRA, version 28.1) is a hierarchical dictionary that is used to code symptoms, signs, and diagnoses, investigations, medical/social history and therapeutic indications. According to the MedDRA, AEs are coded as preferred terms (PTs) using event-related information. MedDRA is organized in a hierarchical structure from the most specific to the most general as follows: lowest level terms (LLTs), preferred terms (PTs), high level terms (HLTs), high level group terms (HLGTs), and system organ classes (SOCs). SMQs are validated, predefined groupings of MedDRA terms developed to consistently retrieve cases related to a specific clinical concept, minimizing variability due to differences in coding practices across countries and reporters.

For this study, using the Line Listing function of EV, data were retrieved for the period 1 January 2021, to 31 December 2025. The period was chosen considering the authorization dates for GLP-1 and GLP-1/GIP RAs for the treatment of weight management (December 2020 for liraglutide, November 2021 for semaglutide, September 2022 for tirzepatide), thus we expected to find in EV database ICSRs related to the use of these medications for this specific indication starting from 2021. In our dataset, duplicate reports were assessed using a two-step procedure. First, reports were screened using the unique case identifier available in EudraVigilance. Second, all ICSRs were systematically cross-checked using patient demographic characteristics (age group and sex), reporting date, suspected study drugs and reported suspected ADRs. ICSRs involving two or more study drugs (i.e., liraglutide, semaglutide, or tirzepatide) were considered ineligible for the comparative analyses and would have been excluded. ICSR selection was restricted to the use of suspected drug for weight management, identified by the presence of at least one MedDRA PT consistent with weight-related therapeutic intent (i.e., the following PTs: “Weight,” “Weight control,” “Weight decreased,” “Overweight” and “Obesity”), and for T2DM, identified by the presence of at least one of the following PTs: “Diabetes mellitus,” “Diabetes mellitus inadequate control,” “Diabetes with hyperosmolarity,” “Diabetic arteritis,” “Diabetic coma,” “Diabetic coronary microangiopathy,” “Diabetic hepatopathy,” “Diabetic hyperglycaemic coma,” “Diabetic metabolic decompensation,” “Diabetic wound,” “Hyperglycaemic crisis,” “Hyperglycaemic hyperosmolar nonketotic syndrome,” “Hyperglycaemic seizure,” “Hyperglycaemic unconsciousness,” “Insulin resistant diabetes,” “Insulin-required type 2 diabetes mellitus,” “Type 2 diabetes mellitus.” For the selection of ICSRs reporting cases of suicide the following PTs captured by the SMQ “Suicide/self-injury” have been included: “Assisted suicide,” “Columbia suicide severity rating scale abnormal,” “Completed suicide,” “Depression suicidal,” “Intentional overdose,” “Intentional self-injury,” “Poisoning deliberate,” “Self-injurious ideation,” “Suicidal behaviour,” “Suicidal ideation,” “Suicide attempt,” “Suicide threat,” “Suspected suicide,” “Suspected suicide attempt.” SOC-based filtering was not applied, because SMQ preferred terms can map to more than one SOC (MedDRA multi-axiality), and restricting to a single SOC could lead to missed cases.

### 2.3 Descriptive analyses

Information on case characteristics was provided for all selected ICSRs and stratified by GLP-1–based therapy and included patients’ age and sex, reporter qualification (HCP vs. non-HCP), principal source country for regulatory purposes (EEA vs. non-EEA), source type of report (spontaneous or not spontaneous), number of AEs, number of suspected drugs, and number of concomitant drugs. Descriptive statistics (mean and standard deviation) were used where applicable.

AEs were also summarized by SOC and stratified by suspected GLP-1–based therapy. Among the five most reported SOCs, the most frequently reported PTs were also identified using a ≥3% frequency threshold. Regarding the seriousness criteria, reports were classified as “serious,” when the described AE(s) was life-threatening, resulted in death, required or prolonged hospitalization, resulted in persistent or significant disability/incapacity, led to a congenital anomaly/birth defect, or represented another medically important condition, or “not serious” (Abraham et al., 2010). Clinical outcomes were instead categorized as “Recovered/Resolved,” “Recovering/Resolving,” “Recovered/Resolved with Sequelae,” “Not Recovered/Not Resolved,” “Fatal,” and “Unknown.” If an ICSR reported two or more adverse events with different outcomes, the outcome reflecting the lowest level of resolution was retained for classification. Reports were classified as fatal when death was reported even though not necessarily related to the suspected drug.

For the subgroup of ICSRs involving suicidal behaviour in the context of weight management indication, case characteristics were described separately for liraglutide, semaglutide, and tirzepatide, including age category, sex, and outcome. Finally, concomitant psychiatric medications were summarized according to their reported indication of use.

### 2.4 Disproportionality analysis

To evaluate the reporting frequency of suicide- and self-injury–related ADRs among ICSRs involving liraglutide, semaglutide, and tirzepatide, the ROR and its 95% CI were calculated. Comparative analyses were performed as pairwise comparisons between liraglutide, semaglutide, and tirzepatide, using the alternative agent in each comparison as the reference group. The ROR was calculated as (a/b)/(c/d), where: “a” is the number of case reports including the adverse event of interest (i.e., suicidal events) reported with the drug of interest (e.g., semaglutide); “b” is the number of case reports not including the adverse event of interest reported with the drug of interest; “c” is the number of case reports including the adverse event of interest reported with the comparator drug (e.g., liraglutide); and “d” is the number of case reports not including the adverse event of interest reported with the comparator drug (Supplementary Table S1). Only reports with weight management as the reported indication were included. Reports classified as “Both” were excluded to ensure a homogeneous study population and consistency between the study cohort and the ROR denominators. At least three reports of the event of interest were required for each drug to perform disproportionality analyses. RORs, 95% CIs, and p-values were calculated using two-sided Fisher’s exact test. The 95% CIs were derived from the exact odds ratio estimate returned by Fisher’s exact test, and no continuity correction was applied. Statistical significance was defined as a two-sided p-value ≤0.05. Data management and analyses were performed using Excel 365 (Microsoft Office) and R (version 4.2.2, R Development Core Team).

## 3 Results

### 3.1 Characteristics of ICSRs

**FIGURE 1:**
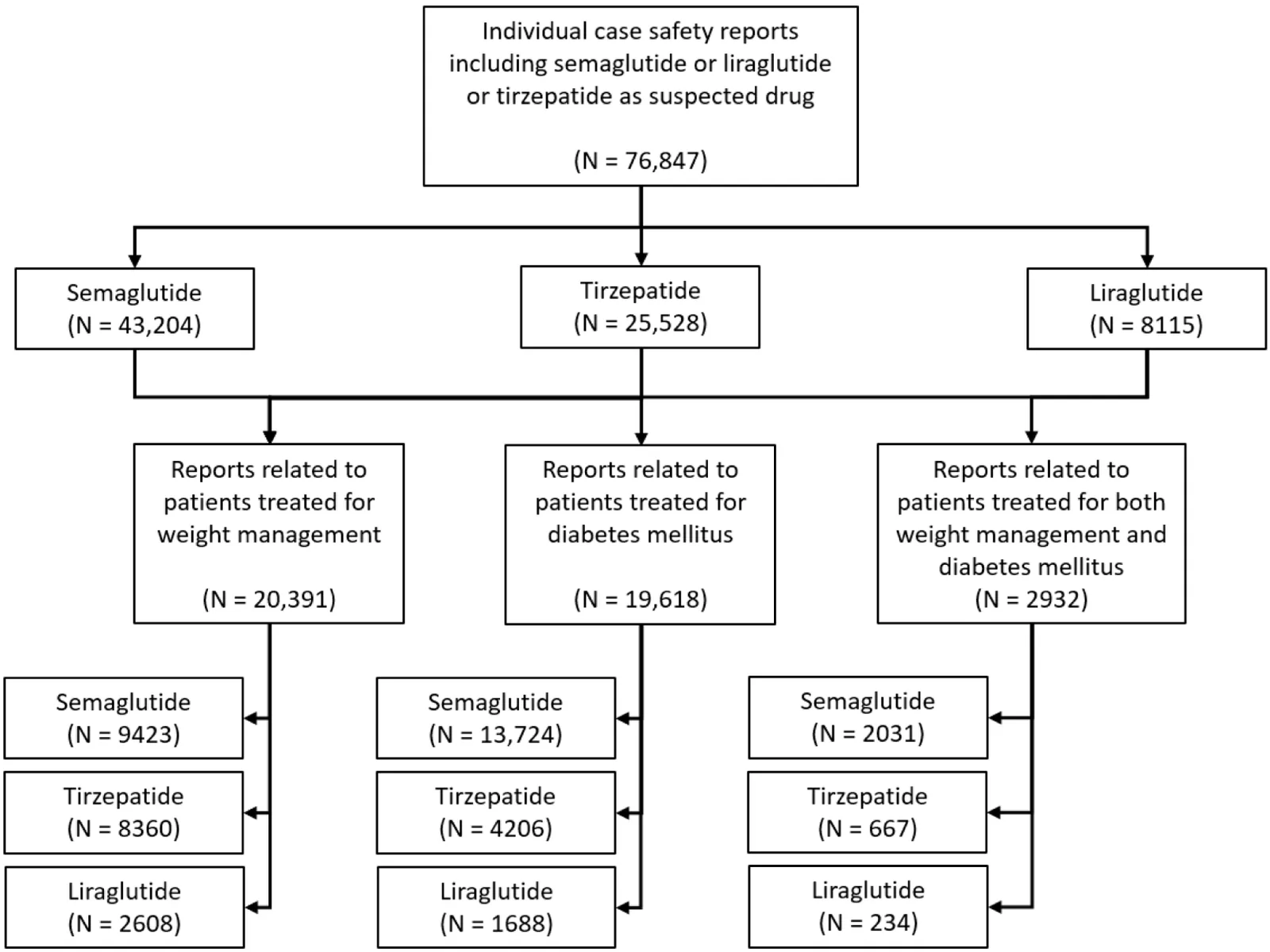
Flowchart of the included individual case safety reports.

Using the EV database, 76,847 ICSRs listing liraglutide, semaglutide, or tirzepatide as suspect drugs were detected during the study period. This initial dataset included all reports in which at least one of the study drugs was listed as suspect, regardless of indication or clinical context. After considering only reports corresponding to weight-related conditions and/or T2DM, 42,941 ICSRs remained eligible for analysis (Figure 1). No duplicate cases were identified after applying the predefined deduplication procedure. In addition, no ICSRs reporting more than one study drug were identified. Among the 33,906 ICSRs excluded from the analysis because they could not be assigned to the indication-defined study population, 31,243 (92.1%) were coded as “Product used for unknown indication.”

Almost 59% of these ICSRs reported semaglutide as suspect drug (n = 25,178), followed by tirzepatide (n = 13,233) and liraglutide (n = 4,530). ICSRs mainly referred to adults aged 18–64 years (57.2%) followed by patients aged 65–85 years (20.4%). Women were reported more frequently than men (62.6% vs. 30.1%), while only a small minority of reports lacked information on sex (7.4%). With respect to indications, weight management and T2DM were reported in broadly similar proportions (47.5% and 45.7%, respectively), whereas a smaller subset of ICSRs listed both conditions (6.8%). All cases were submitted as spontaneous reports. Nearly half of the cases originated from non-HCPs. Most ICSRs were submitted from countries outside the EEA (59.3%). ICSRs reported an average of 3 AEs, 1.31 suspected medications and 2.3 concomitant medications. Regarding case seriousness, the most frequently reported category was other medically important condition (34.9%), followed by hospitalization/prolongation of hospitalization (28.9%) and non-serious reports (32.0%). Reports classified as life-threatening (2.6%), resulting in death (1.3%), resulting in disability (0.3%), or congenital anomaly (0.1%) were uncommon. The distribution of seriousness categories was broadly similar across semaglutide, liraglutide, and tirzepatide (Table 1).

**TABLE 1:** Characteristics of individual case safety reports (ICSRs) listing liraglutide, semaglutide or tirzepatide as suspected drugs.

N. of ICSRs (%)	Liraglutide 4,530 (100.0)	Semaglutide 25,178 (100.0)	Tirzepatide 13,233 (100.0)	Overall 42,941 (100.0)
Age category (%)				
0–1 month	2 (<0.1)	5 (<0.1)	1 (<0.1)	8 (<0.1)
2 months–2 years	1 (<0.1)	6 (<0.1)	3 (<0.1)	10 (<0.1)
3–11 years	4 (0.1)	8 (<0.1)	3 (<0.1)	15 (<0.1)
12–17 years	54 (1.2)	59 (0.2)	11 (0.1)	124 (0.3)
18–64 years	2,773 (61.2)	13,779 (54.7)	8,007 (60.5)	24,559 (57.2)
65–85 years	718 (15.8)	6,159 (24.5)	1888 (14.3)	8,765 (20.4)
More than 85 years	25 (0.6)	211 (0.8)	73 (0.6)	309 (0.7)
Not specified	953 (21.0)	4,951 (19.7)	3,247 (24.5)	9,151 (21.3)
Sex (%)				
Female	3,167 (69.9)	14,959 (59.4)	8,740 (66.0)	26,866 (62.6)
Male	1,110 (24.5)	8,269 (32.8)	3,530 (26.7)	12,909 (30.1)
Not specified	253 (5.6)	1950 (7.7)	963 (7.3)	3,166 (7.4)
Indication of use (%)				
Weight management	2,608 (57.6)	9,423 (37.4)	8,360 (63.2)	20,391 (47.5)
Type 2 diabetes mellitus	1,688 (37.3)	13,724 (54.5)	4,206 (31.8)	19,618 (45.7)
Both	234 (5.2)	2031 (8.1)	667 (5.0)	2,932 (6.8)
Source qualification = Non-HCP (%)	2,346 (51.8)	13,828 (54.9)	5,231 (39.5)	21,405 (49.8)
Source country = Non-EEA (%)	2,433 (53.7)	14,470 (57.5)	8,554 (64.6)	25,457 (59.3)
N. adverse events (mean (SD))	2.95 (2.63)	3.27 (2.76)	2.85 (2.58)	3.11 (2.70)
N. suspected drugs (mean (SD))	1.30 (0.83)	1.31 (0.87)	1.31 (0.77)	1.31 (0.84)
N. concomitant drugs (mean (SD))	1.98 (2.79)	2.53 (3.72)	1.98 (2.90)	2.30 (3.40)
Seriousness				
Results in death	57 (1.3)	340 (1.4)	156 (1.2)	553 (1.3)
Life threatening	119 (2.6)	615 (2.4)	392 (3.0)	1,126 (2.6)
Hospitalization/prolongation of hospitalization	1,199 (26.5)	7,339 (29.1)	3,853 (29.1)	12,391 (28.9)
Results in disability	16 (0.4)	98 (0.4)	2 (<0.1)	116 (0.3)
Congenital anomaly	9 (0.2)	16 (0.1)	7 (0.1)	32 (0.1)
Other medically important condition	1,452 (32.1)	8,620 (34.2)	4,923 (37.2)	14,995 (34.9)
Non-serious	1,678 (37.0)	8,150 (32.4)	3,900 (29.5)	13,728 (32.0)

Across drugs, the SOC “Gastrointestinal disorders” represented the most frequently reported (nausea, vomiting and diarrhoea were the most commonly reported PTs), followed by the SOCs “Injury, poisoning and procedural complications” (off-label use was the most commonly reported PT) and “General disorders and administration site conditions” (fatigue, asthenia and malaise were the most commonly reported PTs) (Tables 2,3). Other commonly reported SOCs included metabolism and nutrition disorders, nervous system disorders, and psychiatric disorders, which showed comparable distributions across drugs.

**TABLE 2:** Distribution of adverse events (AEs) classified by System Organ Class (SOC) reported in the individual case safety reports (ICSRs), stratified by GLP-1 receptor agonist.

System organ class	Semaglutide	Tirzepatide	Liraglutide
N. of adverse events	81,948 (100.0)	37,502 (100.0)	13,276 (100.0)
System organ class, n. (%)			
Blood and lymphatic system disorders	232 (0.3)	172 (0.5)	26 (0.2)
Cardiac disorders	1,247 (1.5)	683 (1.8)	231 (1.7)
Congenital, familial and genetic disorders	60 (0.1)	20 (0.1)	20 (0.2)
Ear and labyrinth disorders	236 (0.3)	137 (0.4)	36 (0.3)
Endocrine disorders	207 (0.3)	106 (0.3)	32 (0.2)
Eye disorders	2,416 (2.9)	616 (1.6)	224 (1.7)
Gastrointestinal disorders	28,449 (34.7)	13,014 (34.7)	4,229 (31.9)
General disorders and administration site conditions	6,575 (8.0)	3,895 (10.4)	1829 (13.8)
Hepatobiliary disorders	1,511 (1.8)	1,389 (3.7)	374 (2.8)
Immune system disorders	287 (0.4)	321 (0.9)	52 (0.4)
Infections and infestations	1,644 (2.0)	849 (2.3)	278 (2.1)
Injury, poisoning and procedural complications	9,239 (11.3)	2,496 (6.7)	1,055 (7.9)
Investigations	4,452 (5.4)	2,477 (6.6)	689 (5.2)
Metabolism and nutrition disorders	5,554 (6.8)	2,489 (6.6)	847 (6.4)
Musculoskeletal and connective tissue disorders	1777 (2.2)	1,095 (2.9)	259 (2.0)
Neoplasms benign, malignant and unspecified	889 (1.1)	306 (0.8)	197 (1.5)
Nervous system disorders	4,780 (5.8)	2,345 (6.3)	819 (6.2)
Pregnancy, puerperium and perinatal conditions	137 (0.2)	82 (0.2)	39 (0.3)
Product issues	628 (0.8)	47 (0.1)	157 (1.2)
Psychiatric disorders	4,223 (5.2)	1,621 (4.3)	602 (4.5)
Renal and urinary disorders	1,151 (1.4)	682 (1.8)	189 (1.4)
Reproductive system and breast disorders	516 (0.6)	384 (1.0)	78 (0.6)
Respiratory, thoracic and mediastinal disorders	1,134 (1.4)	519 (1.4)	180 (1.4)
Skin and subcutaneous tissue disorders	2,187 (2.7)	1,220 (3.3)	456 (3.4)
Social circumstances	266 (0.3)	15 (<0.1)	31 (0.2)
Surgical and medical procedures	1,328 (1.6)	19 (0.1)	200 (1.5)
Vascular disorders	823 (1.0)	503 (1.3)	147 (1.1)

**TABLE 3:** Most frequently reported preferred terms (≥3%) within the five most commonly reported system organ classes (SOCs) in individual case safety reports involving GLP-1 receptor agonists.

System organ class	Preferred term	Semaglutide	Tirzepatide	Liraglutide
Gastrointestinal disorders	Nausea	4,998 (17.6)	1948 (15.0)	849 (20.1)
	Vomiting	3,781 (13.3)	1720 (13.2)	578 (13.7)
	Diarrhoea	2,960 (10.4)	1,694 (13.0)	474 (11.2)
	Impaired gastric emptying	2,957 (10.4)	667 (5.1)	301 (7.1)
	Constipation	2006 (7.1)	774 (5.9)	242 (5.7)
	Abdominal pain	1,488 (5.2)	829 (6.4)	227 (5.4)
	Abdominal pain upper	1,305 (4.6)	609 (4.7)	162 (3.8)
	Dyspepsia	1,061 (3.7)	377 (2.9)	148 (3.5)
Injury, poisoning and procedural complications	Off label use	3,324 (36.0)	900 (36.1)	224 (21.2)
	Inappropriate schedule of product administration	738 (8.0)	107 (4.3)	114 (10.8)
	Wrong technique in product usage process	539 (5.8)	65 (2.6)	36 (3.4)
	Product use issue	473 (5.1)	46 (1.8)	77 (7.3)
	Fall	315 (3.4)	107 (4.3)	49 (4.6)
General disorders	Fatigue	930 (14.1)	366 (9.4)	142 (7.8)
	Asthenia	560 (8.5)	206 (5.3)	99 (5.4)
	Malaise	482 (7.3)	211 (5.4)	93 (5.1)
	Drug ineffective	456 (6.9)	212 (5.4)	186 (10.2)
	Condition aggravated	431 (6.6)	26 (0.7)	56 (3.1)
	Pain	270 (4.1)	159 (4.1)	31 (1.7)
	Illness	198 (3.0)	131 (3.4)	19 (1.0)
Metabolism and nutrition disorders	Decreased appetite	1,686 (30.4)	527 (21.2)	174 (20.5)
	Dehydration	1,118 (20.1)	623 (25.0)	76 (9.0)
	Malnutrition	207 (3.7)	71 (2.9)	19 (2.2)
Nervous system disorders	Dizziness	924 (19.3)	432 (18.4)	174 (21.2)
	Headache	730 (15.3)	324 (13.8)	178 (21.7)
	Loss of consciousness	197 (4.1)	122 (5.2)	35 (4.3)

Preferred terms (PTs) are presented if they accounted for ≥3% of all reported PTs, for at least one drug. For these PTs, the corresponding frequencies and percentages are also shown for the other drugs, regardless of whether they reached the 3% threshold. Percentages were calculated using the total number of reported PTs, for each drug as the denominator.

### 3.2 ICSRs reporting suicidal events

ICSRs describing suicidal events represented only a small fraction of the dataset. Such events were identified in 37 reports involving liraglutide, 141 involving semaglutide, and 47 involving tirzepatide. Most reports concerned adults aged 18–64 years and only a limited number of adolescents were involved, while none were referred to younger age groups. In a subset of cases, particularly among reports involving semaglutide or tirzepatide (22.0% and 31.9%), age was not specified. Women accounted for most reports across all three drugs (81.1%, 68.8%, and 63.8%). Regarding the outcome, the event was described as resolved or resolving in almost 34%–40% of ICSRs. Fatal outcomes were uncommon overall and were observed only in ICSRs reporting liraglutide and semaglutide as suspected drugs (2.7% and 3.5%). A minority of reports did not include information on outcome status (8.1%, 12.1%, and 23.4%) (Table 4).

**TABLE 4:** Characteristics of individual case safety reports in which liraglutide or semaglutide or tirzepatide were listed as suspected drugs for the indication of weight management and including suicidal behaviour as suspected adverse drug reactions.

N. of ICSRs (%)	Liraglutide 37 (100.0%)	Semaglutide 141 (100.0%)	Tirzepatide 47 (100.0%)
Age category			
0–1 month	0	0	0
2 months–2 years	0	0	0
3–11 years	0	0	0
12–17 years	2 (5.4%)	1 (0.7%)	0
18–64 years	32 (86.5%)	100 (70.9%)	32 (68.1%)
65–85 years	0	0	0
More than 85 years	0	9 (6.4%)	0
Not specified	3 (8.1%)	31 (22.0%)	15 (31.9%)
Sex			
Female	30 (81.1%)	97 (68.8%)	30 (63.8%)
Male	7 (18.9%)	37 (26.2%)	10 (21.3%)
Not specified	0	7 (5.0%)	7 (14.9%)
Outcome			
Recovered/resolved	15 (40.5%)	53 (37.6%)	16 (34.0%)
Recovered/resolved with sequelae	0	7 (5.0%)	0
Recovering/Resolving	6 (16.2%)	30 (21.3%)	8 (17.0%)
Not recovered/Not resolved	12 (32.4%)	29 (20.6%)	12 (25.5%)
Fatal	1 (2.7%)	5 (3.5%)	0
Unknown	3 (8.1%)	17 (12.1%)	11 (23.4%)

In 55 ICSRs reporting suicidal events (24.4% of the total), other suspected or concomitant medications indicated for the treatment of psychiatric disorders were reported (Supplementary Table S2). The distribution of suicide- and self-injury-related MedDRA PTs is reported in Supplementary Table S3. Across all treatment groups, suicidal ideation was the most frequently reported PT, accounting for 83.8% of reports with liraglutide, 75.9% with semaglutide, and 85.1% with tirzepatide. Other reported PTs included depression suicidal, suicide attempt, self-injurious ideation, intentional overdose, completed suicide, suicide threat, and suicidal behaviour, each representing a small proportion of cases. Compared with tirzepatide, both liraglutide and semaglutide showed a statistically a significantly higher reporting frequency of ICSRs with suicidal events (ROR 2.54, 95% CI 1.60–4.01 and ROR 2.69, 95% CI 1.91–3.83, respectively) (Table 5).

**TABLE 5:** Reporting odds ratio (ROR) with 95% confidence interval of suicide reports among ICSRs involving liraglutide, semaglutide, or tirzepatide as suspected drugs, with weight management as the reported therapeutic indication.

Comparison	ROR	95% CI	p-value
Liraglutide			
vs. semaglutide	0.95	0.64–1.37	0.85
vs. tirzepatide	2.54	1.60–4.01	4.62 × 10^−5^
Semaglutide			
vs. tirzepatide	2.69	1.91–3.83	5.83 × 10^−10^

## 4 Discussion

The present pharmacovigilance analysis provides updated real-world evidence on the safety profile of GLP-1–based therapies used for T2DM and weight management and the risk of suicide-related events. We found that more than 50% of ICSRs reported semaglutide as suspect drug, followed by tirzepatide and liraglutide, reflecting a higher prescription and use of semaglutide compared to the other GLP-1 RAs (Berning et al., 2025).

The predominance of adult cases observed in our study is likely explained by prescribing patterns, as GLP-1 and GLP-1/GIP RAs are primarily used in adult populations for weight management and T2DM (American Diabetes Associa tion Professional Practice Committee for Diabetes*, 2026). The higher proportion of female reports observed in our dataset may reflect both prescribing patterns in obesity treatment and known sex-related differences in ADR susceptibility, mainly associated with hormonal factors and changes in pharmacokinetics (Scavone et al., 2018; Scavone et al., 2020; Mascolo et al., 2019). Furthermore, the higher proportion of reports submitted by non-HCPs may reflect increased public awareness and media attention surrounding GLP-1–based therapies, potentially contributing to reporting bias.

We found that most AEs belonged to the SOCs “Gastrointestinal disorders,” “Injury, poisoning and procedural complications,” and “General disorders and administration site conditions.” This is in line with data from SmPCs and literature data reporting that these drugs are commonly associated with gastrointestinal symptoms, mainly nausea, injection site reactions, headache, which are usually not serious and do not result in discontinuation of the drug (Filippatos et al., 2014).

Events related to suicide represented less than 0.3% of the total events reported for semaglutide, liraglutide and tirzepatide.

Although these events were rare, their interpretation requires careful consideration of biological plausibility, the available literature, and real-world prescribing patterns.

A potential mechanistic link between GLP-1–based therapies and suicidality has not been definitively established; however, several hypotheses have been proposed. GLP-1 receptors are expressed in key brain regions involved in appetite regulation, reward processing, and emotional control, including the hypothalamus and mesolimbic system. For tirzepatide, the additional activity at the GIP receptor should also be considered when interpreting potential pharmacodynamic effects. Experimental and translational evidence suggests that GLP-1 signalling may modulate dopaminergic pathways and stress-related neurocircuits, potentially influencing mood and behaviour. In particular, alterations in genes involved in dopamine signalling and neuroinflammatory pathways have been hypothesised as potential mediators of psychiatric effects, although these findings remain speculative and insufficient to establish causality, given the multifactorial nature of suicidality (Carmellini et al., 2025; McIntyre et al., 2025). Overall, the available evidence in the current literature appears largely reassuring, with no substantial signals of concern emerging from existing studies. A recent study carried out using data from the EV reported that, although GLP1-RAs exhibit higher reporting of suicide when compared to DPP4 and SGLT2 inhibitors, the criteria for a positive signal are not met (Nakhla et al., 2025). Another recent study based on EV data showed instead a signal for tirzepatide vs. semaglutide for AEs related to injection site reactions (Popa Ilie et al., 2026). A systematic review and meta-analysis of data from RCTs demonstrated that events of suicide and self-harm were rare events in adults treated with GLP-1–based therapies for T2DM and obesity also when compared to placebo. In addition, data from a cohort study involving almost 300,000 adults from Sweden and Denmark revealed no increased risk of suicide deaths, self-harm events, depression, or anxiety with GLP-1 RAs compared to SGLT2 inhibitors (Ueda et al., 2024).

These findings are consistent with the conclusions of regulatory agencies, which do not currently support a causal association between GLP-1 receptor agonists and suicidality. Events of suicide/self-injury are rare occurrences that may be underreported in clinical trials, from which patients at greater psychiatric risk are often excluded.

Pharmacovigilance analyses have suggested a higher reporting probability of suicidal events with liraglutide and semaglutide, as previously reported in EudraVigilance-based studies (Ruggiero et al., 2024), and further differences in safety profiles between GLP-1–based therapies have been observed in real-world settings (Ruggiero et al., 2025).

Both liraglutide and semaglutide showed a higher reporting frequency of suicidal-related adverse events compared with tirzepatide. Many factors could explain this result, including potential differences in cumulative exposure, prescribing patterns, approved indications, and the timing of market adoption that may have influenced spontaneous reporting. In addition, reporting dynamics may have been affected by differences in public and media attention, including social media coverage, as well as by stimulated reporting, all of which should be considered when interpreting these findings (Propfe et al., 2026). Therefore, the observed differences cannot be attributed solely to pharmacological characteristics, such as tirzepatide’s dual GIP/GLP-1 receptor activity, and should be interpreted with caution in light of these potential sources of reporting bias (Taktaz et al., 2024).

Consistent with the absence of a clear causal relationship, a recent meta-analysis including 12 studies found no significant difference in suicidal ideation nor in completed suicide among patients treated with GLP-1–based therapies compared with those receiving other obesity or diabetes medications. However, subgroup analyses showed a higher frequency of suicide among patients treated with liraglutide compared with semaglutide, while no significant difference was observed between tirzepatide and semaglutide. Overall, these findings suggest that current evidence does not support a consistent association between GLP-1–based therapies use and suicidality, while highlighting the need for further randomized controlled trials specifically designed to assess this outcome (Alansari et al., 2025). Therefore, considering that data from the literature do not support a causal association between GLP-1–based therapies and suicidality neither data from regulatory agencies, it is conceivable that the reporting of suicidal-related events in patients receiving GLP-1–based therapies could be the consequence of awareness deriving from safety alerts that induced HCPs and patients to become more aware of a specific adverse event, leading to a temporary spike in reports of that event in spontaneous reporting systems. Indeed, stimulated reporting can distort safety signal detection (McIntyre et al., 2025). In this context, prior experience with antidiabetic therapies has shown that real-world data can provide important insights into safety profiles and prescribing trends over time (Capuano et al., 2013; Rafaniello et al., 2015; Cimmaruta et al., 2016).

### 4.1 Strengths and limitations

This study has several strengths. It is based on a large European pharmacovigilance database, enabling the identification of rare adverse events in a real-world setting across a broad, heterogeneous population. The use of standardised MedDRA terminology and the use of a selected list of PTs derived from the SMQ “Suicide/self-injury” ensured a comprehensive and consistent identification of cases, reducing potential variability related to coding practices. Moreover, the comparative evaluation of different GLP-1–based therapies provides clinically relevant insights into potential differences in reporting patterns, which may improve understanding of their safety profiles in routine clinical practice.

However, some limitations should be acknowledged. First, the inherent nature of spontaneous reporting systems does not allow the estimation of incidence rates or the establishment of causal relationships. Second, ICSRs often lack detailed clinical information, including psychiatric history, socioeconomic factors, and the use of concomitant medications, which may act as important confounders. Although concomitant medications indicated for psychiatric disorders were reported in a minority of suicidal-event ICSRs, the underlying psychiatric conditions and other concomitant diseases potentially confounding this outcome could not be ascertained with certainty. In addition, underreporting and various forms of reporting bias, including stimulated reporting and notoriety bias, may have influenced the observed associations, particularly given the increasing public attention to GLP-1–based therapies. The interpretation of the comparative findings is further limited by differences in market availability and cumulative exposure among the study drugs, particularly the more recent introduction of tirzepatide, which may have affected spontaneous reporting patterns. Differences in time on the market and cumulative exposure between the analysed drugs may have further contributed to the observed disproportionality signals. The absence of information on treatment duration, dosage, and the temporal relationship between drug exposure and event onset limits the ability to fully interpret the clinical relevance of the reported events. Overall, these findings should be interpreted in light of the inherent limitations of disproportionality analyses based on spontaneous reporting systems. The ROR is a measure of reporting disproportionality and should not be interpreted as an estimate of the incidence or causal risk of an adverse event. Furthermore, the presence of concomitant psychiatric medications in a proportion of the reported cases may reflect pre-existing psychiatric conditions or other underlying factors associated with suicidal events, which cannot be adequately accounted for within this type of analysis. Consequently, our findings should be regarded as descriptive and hypothesis-generating, providing supportive pharmacovigilance evidence that complements, rather than replaces, evidence derived from well-designed epidemiological and clinical studies. Lastly, we performed a comparative analysis between GLP-1–based therapies in terms of reporting frequency of suicide-related events, although in the absence of data for a clinically appropriate external comparator, a comparison restricted to these three drugs also cannot assess a possible pattern shared by the entire group.

## 5 Conclusion

This pharmacovigilance study provides updated real-world evidence on suicide- and self-injury–related events. Differences in the relative reporting of these events were observed among liraglutide, semaglutide, and tirzepatide. However, these findings do not establish causality and should not be interpreted as evidence of an increased clinical risk of suicidality associated with any individual GLP-1–based therapy.

Although our data, for their nature, are not able to support any conclusion on a possible increased risk of suicidality associated with GLP-1–based therapies, the presence of a statistically significant disproportionality signals suggest that continued monitoring remains warranted, particularly in populations treated for obesity, who may differ from T2DM patients in terms of demographic and psychiatric profiles.

From a clinical perspective, healthcare professionals should remain vigilant for the occurrence of psychiatric symptoms in patients receiving GLP-1–based therapies, especially in those with pre-existing psychiatric conditions or other risk factors for suicidality.

## Data Availability

The datasets presented in this article are not readily available because the data underlying this study were retrieved from EudraVigilance/ADRreports and are subject to the EudraVigilance access policy and applicable data protection rules. Aggregated results and derived analyses, including reporting odds ratios, are provided in the manuscript. Requests to access the underlying Individual Case Safety Reports should be directed to the EudraVigilance/ADRreports portal: www.adrreports.eu.
