# Supplementary material for: Reporting patterns of suicide- and self-injury-related events involving liraglutide, semaglutide, and tirzepatide: data from the European pharmacovigilance database
Source: Front Pharmacol. 2026 Aug 27;17:1882225. doi: 10.3389/fphar.2026.1882225 (PMC13558095; doi:10.3389/fphar.2026.1882225)
Supplement: Supplementary file 1 [file Supplementaryfile1.docx]

**Table S1.** Contingency tables (2 × 2) used for the calculation of the reporting odds ratios (RORs) comparing semaglutide, liraglutide, and tirzepatide for suicide/self-injury reports among cases with the indication of weight management.

|  | **Suicidal cases** | **Other cases** |
| --- | --- | --- |
| **Liraglutide** | 37 | 2571 |
| **Semaglutide** | 141 | 9282 |
|  | | |
|  | **Suicidal cases** | **Other cases** |
| **Liraglutide** | 37 | 2571 |
| **Tirzepatide** | 47 | 8313 |
|  | | |
|  | **Suicidal cases** | **Other cases** |
| **Semaglutide** | 141 | 9282 |
| **Tirzepatide** | 47 | 8313 |
|  | | |

**Table S2.** Suspected and concomitant medications reported in suicide/self-injury cases. The table indicates whether each case included at least one medication commonly used in psychiatric practice, identifies the corresponding psychiatric medication(s), and reports their primary psychiatric indication(s), where applicable.

| **Case n.** | **GLP1-receptor agonist** | **Psychiatric drug** | **Indication** |
| --- | --- | --- | --- |
| 1 | Liraglutide | escitalopram, citalopram | Depression/anxiety, Depression |
| 2 |  | venlafaxine | Depression/anxiety |
| 3 |  | lisdexamfetamine | ADHD |
| 4 |  | paroxetine | Depression/anxiety |
| 5 |  | paroxetine | Depression/anxiety |
| 6 |  | venlafaxine, quetiapine | Depression/anxiety, Schizophrenia/bipolar disorder |
| 7 |  | venlafaxine | Depression/anxiety |
| 8 |  | sertraline, lisdexamfetamine | Depression/anxiety, ADHD |
| 9 |  | fluoxetine | Depression |
| 10 |  | trazodone | Depression/insomnia |
| 11 |  | fluoxetine, quetiapine, pregabalin | Depression, Schizophrenia/bipolar disorder, Anxiety/neuropathic pain |
| 12 | Semaglutide | quetiapine, aripiprazole | Schizophrenia/bipolar disorder |
| 13 |  | escitalopram, citalopram | Depression/anxiety, Depression |
| 14 |  | citalopram | Depression |
| 15 |  | escitalopram, citalopram, lorazepam | Depression/anxiety, Depression, Anxiety |
| 16 |  | lithium | Bipolar disorder |
| 17 |  | escitalopram, citalopram, lisdexamfetamine | Depression/anxiety, Depression, ADHD |
| 18 |  | bupropion | Depression/smoking cessation |
| 19 |  | sertraline, trazodone | Depression/anxiety, Depression/insomnia |
| 20 |  | escitalopram, citalopram, bupropion | Depression/anxiety, Depression, Depression/smoking cessation |
| 21 |  | sertraline, venlafaxine | Depression/anxiety |
| 22 |  | paroxetine | Depression/anxiety |
| 23 |  | escitalopram, citalopram | Depression/anxiety, Depression |
| 24 |  | lithium | Bipolar disorder |
| 25 |  | carbamazepine | Bipolar disorder/epilepsy |
| 26 |  | quetiapine | Schizophrenia/bipolar disorder |
| 27 |  | duloxetine | Depression/anxiety/neuropathic pain |
| 28 |  | fluoxetine | Depression |
| 29 |  | duloxetine | Depression/anxiety/neuropathic pain |
| 30 |  | sertraline | Depression/anxiety |
| 31 |  | quetiapine | Schizophrenia/bipolar disorder |
| 32 |  | venlafaxine | Depression/anxiety |
| 33 |  | fluoxetine | Depression |
| 34 |  | escitalopram, citalopram | Depression/anxiety, Depression |
| 35 |  | sertraline, quetiapine, methylphenidate | Depression/anxiety, Schizophrenia/bipolar disorder, ADHD |
| 36 |  | citalopram, bupropion, aripiprazole | Depression, Depression/smoking cessation, Schizophrenia/bipolar disorder |
| 37 |  | alprazolam, zolpidem | Anxiety, Insomnia |
| 38 |  | sertraline | Depression/anxiety |
| 39 |  | lisdexamfetamine | ADHD |
| 40 |  | duloxetine | Depression/anxiety/neuropathic pain |
| 41 |  | venlafaxine | Depression/anxiety |
| 42 |  | duloxetine | Depression/anxiety/neuropathic pain |
| 43 |  | fluoxetine | Depression |
| 44 |  | citalopram | Depression |
| 45 |  | diazepam, valpro | Anxiety, Bipolar disorder/epilepsy |
| 46 |  | diazepam, valpro | Anxiety, Bipolar disorder/epilepsy |
| 47 |  | sertraline | Depression/anxiety |
| 48 |  | pregabalin | Anxiety/neuropathic pain |
| 49 | Tirzepatide | diazepam | Anxiety |
| 50 |  | duloxetine | Depression/anxiety/neuropathic pain |
| 51 |  | duloxetine, lamotrigine | Depression/anxiety/neuropathic pain, Bipolar disorder/epilepsy |
| 52 |  | venlafaxine | Depression/anxiety |
| 53 |  | bupropion | Depression/smoking cessation |
| 54 |  | fluoxetine, mirtazapine | Depression |
| 55 |  | sertraline | Depression/anxiety |

**Table S3.** Distribution of MedDRA Preferred Terms (PTs) related to suicide and self-injury among reports associated with semaglutide, liraglutide, and tirzepatide.

| **GLP1-receptor agonist** | **Suicide-related Preferred Term** | **N. of cases (%)** |
| --- | --- | --- |
| Liraglutide  (N=37, 100.0%) | Suicidal ideation | 31 (83.8%) |
|  | Depression suicidal | 2 (5.4%) |
|  | Self-injurious ideation | 2 (5.4%) |
|  | Intentional overdose | 1 (2.7%) |
|  | Completed suicide | 1 (2.7%) |
| Semaglutide  (N=141, 100.0%) | Suicidal ideation | 107 (75.9) |
|  | Depression suicidal | 9 (6.4) |
|  | Suicide attempt | 8 (5.7) |
|  | Self-injurious ideation | 5 (3.5) |
|  | Completed suicide | 5 (3.5) |
|  | Intentional overdose | 4 (2.8) |
|  | Suicide threat | 2 (1.4) |
|  | Suicidal behaviour | 1 (0.7) |
| Tirzepatide  (N=47, 100.0%) | Suicidal ideation | 40 (85.1) |
|  | Depression suicidal | 6 (12.8) |
|  | Suicide attempt | 1 (2.1) |
